# Methylnaltrexone and Naloxone for Opioid-induced Constipation in the Critical Care Setting

**DOI:** 10.7759/cureus.6829

**Published:** 2020-01-31

**Authors:** Harneel S Saini, Zara Alvi, Bavandeep Singh, Basant Elsharkawy, Muhammad Yasir

**Affiliations:** 1 Neurology, Allegheny General Hospital, Pittsburgh, USA; 2 Internal Medicine, Allegheny Health Network, Pittsburgh, USA; 3 Pharmacy, Lake Erie College of Osteopathic Medicine, Bradenton, USA; 4 Pharmacy, Lake Erie College of Osteopathic Medicine, Cleveland, USA; 5 Internal Medicine, King Edward Medical University, Mayo Hospital, Lahore, PAK

**Keywords:** oic, constipation, opioid

## Abstract

Opioid antagonists in the ICU are often a last-line medication given to patients with opioid-induced constipation. Traditionally, patients have been administered nonopioid-based bowel regimens such as senna, peg, and docusate to treat constipation. Despite the obvious need to treat acute pain with opioids, side effects such as constipation can lead to multiple gastrointestinal (GI) complications such as bowel perforation and even death. Specifically, opioid-induced constipation (OIC) can be very difficult to treat. We examine naloxone and methylnaltrexone (MNTX) assessing GI complications and OIC as well as present a patient case which highlights the importance of treating OIC. We also evaluate the superior reversal agent of choice when treating OIC in the critical care and stepdown unit settings.

## Introduction

Opioids are primarily used to relieve pain but can affect mood, appetite, blood pressure and even breathing. Opioid substrates bind to an opioid receptor (μ, δ, and κ) found in the gastrointestinal (GI) tract as well as the central and peripheral nervous system. Opioid-induced constipation (OIC) is the most commonly encountered adverse side effect of opioid use in critically ill patients [1]. Opioid-induced constipation is thought to be a dose-related, peripheral receptor mediated, side effect and can occur in up to 85% of the patients taking opioids [2]. Consensus definition of OIC is change in baseline bowel and defecation patterns including decreased frequency, harder stool consistency, or straining. Studies about the most suitable opioid antagonist as the agent of choice are still limited [1]. Because opioid receptors are spread throughout the body their effect on multiple endogenous physiological processes is associated with unwanted side effects including bowel dysfunction. Opioids are known to decrease the release of excitatory neurotransmitters in the enteric nervous system interfering with coordinated muscle contractions, decreased intestinal secretions, colonic spams, and increased anal sphincter tone. All these combined effects lead to constipation. At the same time, opioids cause decreased esophageal and increased antral tone causing reflux and delayed gastric emptying, increasing the risk of aspiration. Consecutive nausea and vomiting may interfere with absorption of other drugs and contribute to deconditioning especially in severely ill patients [3]. On the contrary, naloxone and methylnaltrexone (MNTX) are opioid antagonists that bind antagonistically to μ receptors thus minimizing the side effects of opioids such as constipation. Naloxone and MNTX can be administered both enterally and parenterally. Advantageously, MNTX works by binding to same μ receptors in the gut as naloxone does but its inability to cross the blood-brain barrier offers reversal of the unwanted GI side effects without reversal of the analgesic effects of opioids. Theoretically, MNTX is a quarternary μ-opioid receptor antagonist that, unlike naltrexone (NTX) or naloxone has limited ability to cross the blood-brain barrier. Therefore, with oral, intravenous, or topical administration it should not impair centrally mediated analgesic effects of opioids [3].

## Case presentation

**Figure 1:**
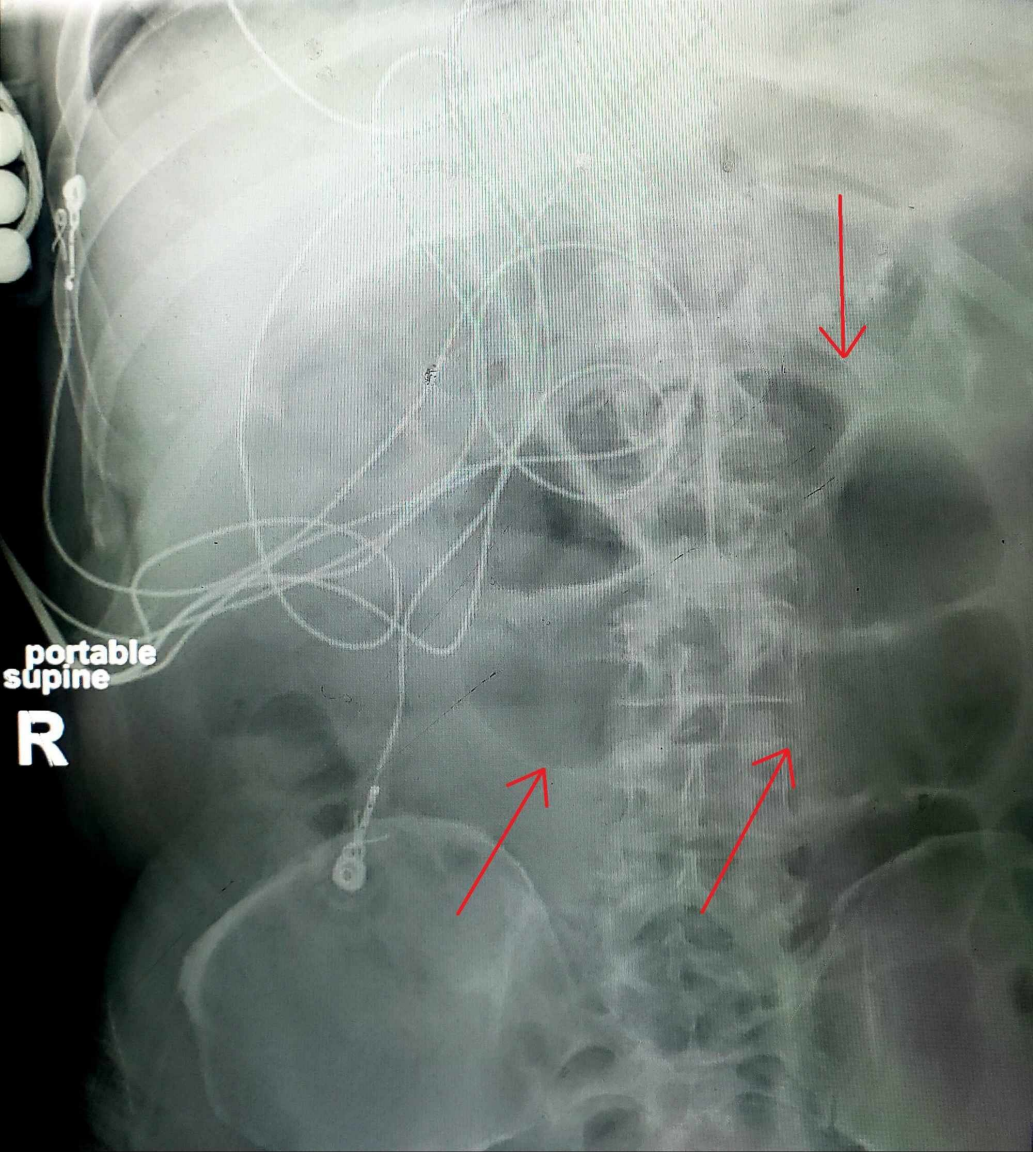
Abdominal X-ray. Red arrows show moderate stool burden with bowel distension.

A 52-year-old male with a past medical history of hypertension, and type 2 diabetes presented to our ED as a level 2 trauma after a ground level mechanical fall causing him to land on his right hip. On presentation, he had a Glasgow Coma Scale of 15; he was awake, alert, and oriented to person, place, and time. No open lesions or wounds were appreciated. His lungs were clear to auscultation bilaterally and were neurovascularly intact in all four extremities with 2+ peripheral pulses. His abdominal exam was unremarkable, soft nondistended, nontender without rebound and guarding. He stated that his last bowel movement (BM) was the night before presentation. His right lower extremity was shortened and externally rotated without any other obvious deformities. Neuro exam was grossly intact 5/5 strength throughout with sensation to pinprick and vibration intact. No abrasions, bleeding, or bruises were noted. He complained of 10 out of 10, severe pain localizing to his right hip which worsened with movement. He denied dizziness and loss of consciousness prior to the fall. Vitals at the time of presentation were consistent with a blood pressure of 154/95, heart rate of 108, respiratory rate of 16, saturating at 85% on 4 L of oxygen on nasal cannula. He was found to be hyponatremic, hyperchloremic with metabolic acidosis and without an elevation in hepatic enzymes (see Table 1). He also had an acute kidney injury with an elevated creatinine and BUN, anemic with a hemoglobin of 8.5, and an INR of 1.3. Chest X-ray showed pulmonary congestion/edema with cardiomegaly without a previous echocardiogram on file. Imaging of this right hip showed an acute angulated right, nondisplaced femoral neck fracture. The patient was put on a nonrebreather mask at 10 L/min and sent to the Complex Medical Care Unit (CMCU) with an orthopedic consultation. On arrival to the CMCU, he was transitioned to 6 L of nasal cannula with plans of orthopedic intervention the following day. Labs drawn on the floor were significant for a proBNP of 11,800. His acute hypoxic respiratory failure was thought to be related to a new found congestive heart failure (CHF) exacerbation and he was diuresed accordingly with intravenous furosemide with specific nursing instructions for strict documentation regarding fluid inputs and outputs via a foley catheter. Management of his acute kidney injury was appropriately treated with a nephrology consult. The patient was anticoagulated with heparin 5000 units every eight hours. Pain control was adequately achieved with opioids, initially 2 mg of morphine for intermediate pain and 4 mg of morphine for severe pain as needed every four hours and he was started on a senna and docusate bowel regimen. The next day, our patient was taken to the operating room placed under endotracheal induction anesthesia and subsequently desaturated to 68% requiring the surgery to be aborted. It was deemed the safest option would be to attempt surgery at a later time after he was more medically optimized and as delaying the surgery would not alter his ultimate treatment. Anesthesia was reversed and he was extubated to 6 L of oxygen via nasal cannula. The patient returned to the CMCU alert and oriented. During the interim course leading up to his total right hip arthroplasty seven days later, he was hemodialysed a total of two times, weaned to 2 L of oxygen via nasal cannula with marked improvement in his chest X-ray. He tolerated the surgery very well and returned to the CMCU alert and oriented on 1 L of oxygen via nasal cannula and was subsequently weaned off nasal cannula to room air. His pain was adequately managed throughout the rest of his hospital course with parenteral fentanyl and oral oxycodone as needed. A few days after his surgery, he could not tolerate a regular carbohydrate diet as he would become very nauseous and then vomit. He also had not had a BM after his surgery, four days prior and felt very bloated. An abdominal X-ray was obtained to evaluate stool burden which showed findings consistent with moderate colonic distension most suggestive of colonic ileus favored over distal small bowel obstruction (see Figure 1). In order to relieve the stool burden we administered polyethylene glycol (17 g x two doses), senna (17.2 mg x three doses), senna-docusate (8.6-50 mg x three doses) orally and two tap water enemas and bisacodyl (10 mg x one dose) rectally without avail. His INR was gradually trending upwards as he was nutritionally deprived; we trended his hemoglobin and kept a close eye on his vitals while administering a one-time dose of vitamin K which resolved the issue. With no success in his BMs and increasing stool burden it was determined that his constipation was opioid induced and he was treated with intravenous MNTX (8 mg). Within three hours of administration, the patient had a huge BM which instantly resolved both his nausea and vomiting along with his bloating. He had received 204 milliequivalents of morphine over the course of his hospital stay. Interestingly, the one-time dose of MNTX did not reverse his analgesic effect from the opioids he was receiving for pain management and resolved his constipation rapidly. He was able to tolerate a regular diet once again and was discharged shortly afterwards to a rehabilitation facility for ongoing needs for physical therapy for his hospital stay induced deconditioning.

**Table 1:** Labs prior to treatment. mm/L, millimol per liter; mg/dL, milligrams per deciliter; pg/mL, picogram per milliliter; g/dL, grams per deciliter; BUN, blood urine nitrogen; Cr, creatinine; ProBNP, brain natriuretic peptide; Hgb, hemoglobin; INR, international normalized ratio

Na	129 mm/L
K	3.5 mm/L
Cl	91 mm/L
CO2	20 mm/L
BUN	52 mg/dL
Cr	8.27 mg/dL
ProBNP	11800 pg/mL
Hgb	8.5 g/dL
INR	1.3

## Discussion

Opioid-induced constipation is thought to be primarily a dose-related peripheral receptor mediated side effect and can occur in up to 85% of the patients taking opioids. Single large doses of oral naloxone have been shown to be effective for reversal of opioid-induced constipation; however, they have been noted to accompany unwanted adverse effect of analgesia reversal. A double-blinded, randomized, placebo-controlled study was done with nine patients who were given naloxone vs. placebo and outcomes including stool frequency along with symptoms of constipation and amount of analgesia required for pain control were recorded. Results showed all patients with naloxone had improvement in bowel frequency and three patients experienced reversal of analgesia, one of whom had complete reversal of analgesia on day 2 of treatment. Interestingly the study showed that patients using higher doses of opioids appeared to be more vulnerable to analgesic reversal effects of oral naloxone and that this effect persisted despite dividing oral naloxone into very low doses relative to the total dose of opioid used. Additionally, all subjects who were sensitive to analgesic reversal effects did not return to baseline opioid doses until several days after stopping naloxone as a finding likely concerning for possible long-term effect of naloxone on the bowels [2]. Oral naloxone blocks opioid receptors in the intestine and has limited bioavailability due to hepatic first pass metabolism [4]. A very interesting placebo control study looked at the effect of enteral naloxone on aspiration pneumonia, gastric tube reflex, and time until laxation in 84 mechanically ventilated ICU patients. This prospective, randomized double-blinded study provided good quality evidence that naloxone and other opioid antagonists may be simple and preventive measures for reducing the frequency of pneumonia and gastric reflux [5]. Another prospective study looked at enteral naloxone and its effect on analgesia and OIC. The study included 22 patients and reported improvement in time until laxation and decrease in laxative use in the naloxone group with negligible analgesic reversal [4] .

Studies regarding the most suitable opioid antagonist as the agent of choice are still limited. A single-center retrospective review was done on 100 patients in the MICU on continuous fentanyl infusion to evaluate the efficacy of enteral NTX vs. subcutaneous MNTX in the management of OIC (no BM for 72 h for the purpose of the study). Primary outcome was time to first BM, while secondary outcomes included total numbers of BMs within 48 hours, opioid requirements after use of either agent and changes in heart rate, mean arterial pressure and level of sedation after the use of either agent. Results showed no statistical difference in median times for first BM on both agents, which occurred at a median of 72 h, along with stable secondary outcomes. Interesting findings from the review showed that even though MTNX is subcutaneous it was neither affected by use of vasopressors nor did it appear to induce opioid reversal as evidenced by stable vitals and opioid requirements. Additionally, it was found that if no BM was noted within 24 h of NTX administration, MNTX could be used as a potentially cost-saving option. Of note was the fact that there was no clinically significant difference in both groups in terms of gastric residual or evidence of bowel perforation [1]. A retrospective study evaluated the resolution of GI stasis in 15 nonsurgical ICU patients who had received opioids as pain control agents. They compared subcutaneous MTNX versus conventional rescue therapy (sodium picosulfate and two glycerin suppositories) in patients who had not had a BM within 72 h despite conventional laxation therapy with senna and sodium docusate. They concluded 86% of the patients who had received MNTX had laxation within 24 h of administration while none had laxation in the conventional rescue therapy group (P < 0.001). They further reported the median difference laxation time between the two groups was 3.5 days (P < 0.001) with 100% of the patients treated with MNTX progressed to full enteral feeding shortly after [6]. In another study 16 patients were retrospectively evaluated for time till feeding restarted, doses of naloxone, and time to BM. The study concluded 93% of the patients had passed BMs during the study period with a median time to BM of 1 day doses to BM of 3. They further reported 78% of their patients who were not receiving tube feeds restarted continuous tube feeds after naloxone initiation [7]. The optimal naloxone dosing frequency and amount for OIC is still not elucidated as only 2% after first pass of the naloxone remains in the vasculature. The study concluded that naloxone requires more frequency and higher doses to be a good candidate for OIC when compared to the more cost-efficient MNTX [7]. In our case the patient had a prolonged hospital stay, increased use of hospital resources, and worsening metabolic and GI symptoms as the patient could not keep down his oral medication because of nausea and vomiting. Metabolically his INR began to climb upwards as he was malnourished and he required further treatment to prevent any hematological complication.

## Conclusions

Opioid-induced constipation (OIC) is a common and often quickly resolving side effect of opioid use. However in critically ill patients, it can progress insidiously. GI complications are on the more severe side of the spectrum and should be adequately managed. However, keeping a good balance between analgesia and the side effects of opioid administration is very crucial for patient satisfaction. Across the studies there seemed to be a consensus that MNTX an opioid antagonist which does not cross the blood-brain barrier would be a better cost-effective option. In our opinion, further good quality research is needed and should be directed towards cost-effectiveness of administering opioid antagonists vs continuing with conventional laxation therapy for patients who are already being treated with opioids. The risks and benefits should also be assessed with regard to cost due to increased hospital stay and utilization of extra resources like imaging, staff, and cost of medications. MNTX can be a good first-line option for OIC in critically ill patients.
